# Supplementary material for: Spirituality, Conspiracy Beliefs, and Use of Complementary Medicine in Vaccine Attitudes: A Cross-Sectional Study in Northern Italy
Source: Int J Environ Res Public Health. 2025 Mar 11;22(3):413. doi: 10.3390/ijerph22030413 (PMC11942485; doi:10.3390/ijerph22030413)
Supplement: Supplementary file 1 [file ijerph-22-00413-s001.zip › ijerph-3442357-supplementary.pdf]

S1 Statistical analyses using the Mann-Whitney U and Kruskal-Wallis tests showed no significant variations in spirituality among individuals who have been vaccinated against COVID-19 a different number of times: one time (2.6%), two times (18.8%), three times (57.2%), more than three times (13.4%), or none (7.9%). Similarly, no differences in GrAw-7 scores were found between those who supported vaccinating their children (56.1%), those who supported vaccinating their children (7.4%), and those who were indifferent (36.5%). The same outcome was observed when comparing participants who had received the influenza vaccine (18.6%), those who did not (73.8%), and those who were unaware of their status (7.8%). These findings are presented in Supplementary Figure S1. As a result, our subsequent examinations of spirituality focused on the combined score of the perceived risks associated with COVID-19 vaccination and mandatory childhood immunisation.

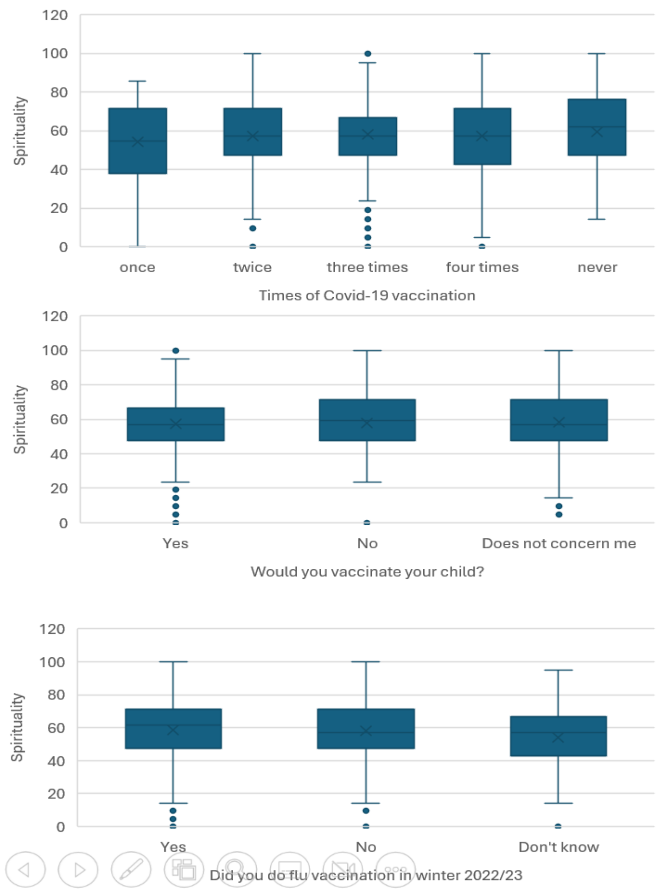

Figure S1. Box plots illustrate the distribution of spirituality scores across three vaccination-related variables. The top panel shows spirituality scores by the number of COVID-19 times, covid-19 vaccination was done, ranging from "once" to “four or more” and "never.” The middle panel displays spirituality scores based on willingness to vaccinate one's child, with responses of "Yes," "No," and "Does not concern me." The bottom panel illustrates spirituality scores in relation to flu vaccination status during the winter of 2022/23, with options of "Yes," "No," and "Don't know." Each box plot indicates the median, interquartile range, and outliers, allowing for a visual comparison of spirituality across different vaccination attitudes and behaviors.

S2. For the harmfulness score of mandatory childhood vaccination (Table S2), the quadratic terms were not statistically significant, despite conspiracy thinking, which substituted the linear term. This factor did not change the model quality and parameters; thus, we left the untransformed linear conspiracy regressor in the model. The model was calculated using $n = 1{,}369$ cases and had an overall corrected R^2 of 0.420. The standardised residuals were not normally distributed. Fourteen standardised residuals of > 3. Excluding these outliers, the significant variables remained the same, and the coefficients changed only slightly. The model without outliers fitted slightly better ($R^2 = 0.450$), but the standardised residuals remained not normally distributed with a mean of 0. Thus, we stopped further analysis of this dependent variable.

Table S1. Predictors of the harmfulness of mandatory childhood vaccination in South Tyrol, Italy, in March 2023 in multivariate linear regression analyses.

**Predictors of harmfulness of mandatory childhood vaccination (*n* = 1,369)**	**Correlation R^2 = 0.420**		
	**Regression coefficient b**	**[95% CI]**	***p*–value**
Constant term	8.586	[7.385; 9.787]	<0.001
Conspiracy thinking	0.191	[0.157; 0.224]	<0.001
Due to the pandemic, I support mandatory childhood vaccination more now			n.s.
Due to the pandemic, I support mandatory childhood vaccination less now	5.703	[4.984; 6.423]	<0.001
CAM consultation			n.s.
Better economic situation			n.s.
Worse economic situation	0.641	[0.162; 1.120]	0.009
Don't know about the economic situation	2.298	[1.079; 3.517]	0.001
Low educational status			n.s.
Vocational school			n.s.
University degree			n.s.
Trust in institutions	−0.144	[−0.167; −0.120]	<0.001
GP consultation	−0.927	[−1.453; −0.401]	0.001
Spirituality	0.013	[0.003; 0.024]	0.016
Trust in vaccination staff			n.s.
Other/ more than one mother tongue	0.909	[0.056; 1.761]	0.037

p–values for the significant contribution of the independent variables to the model. Abbreviations: CAM, complementary and alternative medicine; CI, confidence interval; GP, general practitioner.
